# Acute Increases in Intracellular Zinc Lead to an Increased Lysosomal and Mitochondrial Autophagy and Subsequent Cell Demise in Malignant Melanoma

**DOI:** 10.3390/ijms22020667

**Published:** 2021-01-11

**Authors:** Emil Rudolf, Kamil Rudolf

**Affiliations:** Department of Medical Biology and Genetics, Faculty of Medicine in Hradec Kralove, Charles University, Zborovska 2089, 500 03 Hradec Kralove, Czech Republic; kamil.rudolf@fnhk.cz

**Keywords:** zinc, melanoma, autophagy, cell death, mitochondria, lysosomes

## Abstract

Changes in zinc content and dysregulated zinc homeostatic mechanisms have been recognized in several solid malignancies such as prostate cancer, breast cancer, or pancreatic cancer. Moreover, it has been shown that zinc serum and/or tissue levels are altered in melanoma with varying effects on melanoma development and biology. This study was conducted to explore the effects of acute increases of intracellular zinc in a set of melanoma tissue explants obtained from clinical samples. Measurements of their zinc content showed an extant heterogeneity in total and free intracellular zinc pools associated with varying biological behavior of individual cells, e.g., autophagy levels and propensity to cell death. Use of zinc pyrithione elevated intracellular zinc in a short time frame which resulted in marked changes in mitochondrial activity and lysosomes. These alterations were accompanied by significantly enhanced autophagy flux and subsequent cell demise in the absence of typical apoptotic cell death markers. The present results show for the first time that acutely increased intracellular zinc in melanoma cells specifically enhances their autophagic activity via mitochondria and lysosomes which leads to autophagic cell death. While biologically relevant, this discovery may contribute to our understanding and exploration of zinc in relation to autophagy as a means of controlling melanoma growth and survival.

## 1. Introduction

Cutaneous melanoma is a type of malignant disease with relatively low but worldwide increasing incidence. While treatable in early stages by surgical resection, advanced forms of this condition show increased propensity for biological aggressiveness, systemic spread, and chemoresistance, resulting in treatment failure and high mortality rates among patients. Melanoma cells in these stages present with a heterogeneous genetic background characterized by a high prevalence of somatic mutations and the functional plasticity, favoring an aggressive and invasive phenotype associated with developed chemoresistance [1]. Intensive research over the past decades helped in identification of key molecular hallmarks of melanoma which allowed its classification into four subgroups: tumors with mutant (1) *BRAF* gene, (2) *NRAS* gene, (3) *NF1* gene, and (4) so-called triple wild type melanomas [2]. The mentioned mutated genes as well as a number of involved epigenetic events occurring in melanoma cells contribute to the dysregulation of several signaling pathways comprising hyperactivated BRAF-MEK, PI3K/PTEN, or c-KIT cascades [3]. In addition, other processes and cellular compartments have been found to contribute to the ultimate aggressive phenotype of melanoma cells [4,5,6], with the one currently intensively investigated being autophagy.

Autophagy is a universal process whereby eukaryotic cells degrade and recycle their contents in a selective or non-selective way using currently three recognized autophagy forms; macroautophagy, microautophagy, and chaperon-mediated autophagy [7]. In the cell, autophagy is controlled by coordinated activities of more than 30 autophagy related genes whose individual roles as well as regulation were repeatedly reviewed [8,9]. Autophagy and its dysregulation have been reported to play dual roles in cancer development where its tumor-suppressive activities are recognized at the beginning of malignant transformation whereas its tumor-promoting function is thought to occur at later stages [10,11].

The role of autophagy during melanoma progression is still not clear, however, available knowledge supports an active involvement of autophagy in melanomagenesis and ultimate melanoma dissemination. In particular, in early melanomagenesis the tumor-suppressive role of autophagy is repressed as evident from several studies investigating the expression levels of autophagy-specific proteins (Beclin-1 and LC3B) [12] and genes (*ATG5*) [13]. Furthermore, the existence of a reduced autophagic flux in primary melanoma cells as compared with melanocytes was also confirmed with help of an autophagy inhibitor chloroquine [14].

Accordingly, the presence of a higher autophagic flux in metastatic melanoma cells as compared to primary melanoma and melanocytes is regarded as one of the key transitions towards aggressive melanoma phenotype [14]. Such a conclusion based on in vitro experiments is further supported by findings of higher expression of autophagic LC3B and Beclin-1 proteins in samples of patients with advanced/metastatic melanoma as compared to non-invasive, primary tumors [15,16,17].

Zinc is an essential microelement involved in many cellular processes including cell growth, gene expression, signal transduction, redox balance, as well as cell death [18]. Zinc is present in all body tissues and organs, however, its intracellular content, forms and distribution differ in individual cells with respect to their actual need [19]. Zinc in human cells originates from external sources and its management typically involves influx, efflux, buffering, and muffling mechanisms. These comprise of but are not limited to zinc transport proteins including the ZnT family of transporters [20], the ZIP family of transporters [21] and metallothioneins (MTs) [22]. This network ensures the stability of intracellular zinc levels via zinc-specific transport and sequestering to particular subcellular compartments and zinc-binding proteins. Only a minor pool of intracellular zinc is allowed to be in free form and thus readily available for particular biological effects. Accordingly, acute or chronic changes in mostly free intracellular zinc levels associate with several known pathologies such as immunodeficiency [23], respiratory deficiencies [24], defective growth and wound repair [25], decline in sensory acuity as well as neurodegeneration [26], and development of neoplasia [27]. To this extent, compromised zinc homeostasis is linked to many human neoplasias such as prostate, breast, pancreas, lung, colorectal, gastric, and skin [28].

The role of zinc in the progression and survival of melanoma cells, particularly in relation to autophagy remains unsettled. Zinc may influence autophagic flux in a number of ways which are likely to differ in individual malignant cells. Thus, the present study was prepared to address the effects of acute zinc overload on autophagy, survival, and death of melanoma cells obtained from clinical samples of patients in advanced stages of this condition.

## 2. Results

### 2.1. Proliferation and Zinc Content in Human Melanoma Cells and Melanocytes

**Figure 1:**
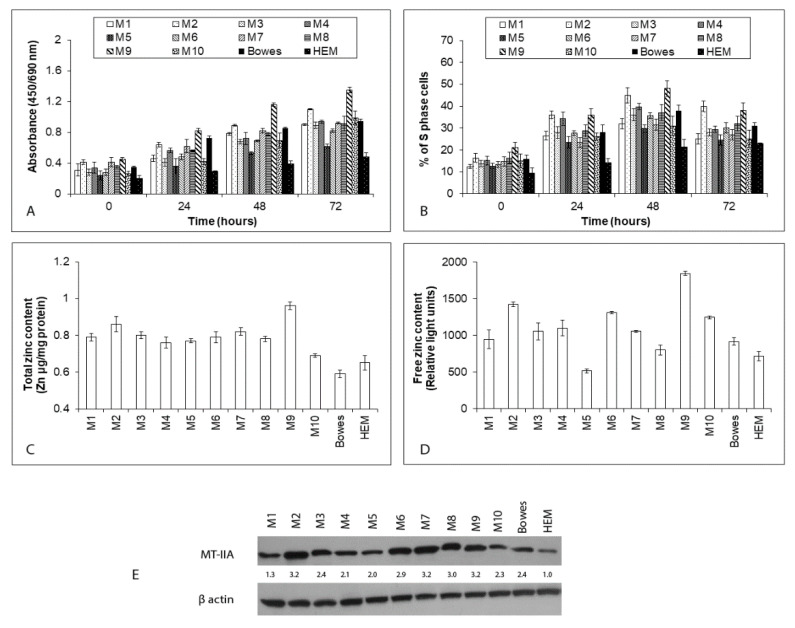
The proliferation, zinc content, and metallothionein II A (MT-IIA) expression in human explant melanoma cells, Bowes cell line and normal human melanocytes HEM. Established explant human melanoma cultures (labeled M1–M10), human melanoma cell line Bowes and normal human melanocytes HEM were maintained upon standard laboratory conditions and their proliferation was determined by (**A**) colorimetric WST-1 assay measuring the cleavage of tetrazolium salt WST-1 by mitochondrial succinate dehydrogenases in viable cells and by (**B**) measuring the proportion of S-phase cells via EdU-specific fluorescence. Values represent means ± SD of at least three experiments. (**C**) Total zinc content in human explant melanoma cells (M1–M10), Bowes cell line and normal human melanocytes HEM. Zinc content was determined by absorption spectrometry. (**D**) Free (labile) zinc content in human explant melanoma cells (M1–M10), Bowes cell line and normal human melanocytes HEM as measured by microfluorometry of the zinc-specific dye Newport Green diacetate. Values represent means ± SD of at least three experiments. (**E**) The expression of metallothionein II-A (MT-IIA) in melanoma cell lysates as determined by immunoblotting analysis. The numbers in the blot image refer to fold increase or decrease in the density of the particular protein compared to the density of the same protein in HEM cells. Shown is one typical result of at least four experiments.

Melanoma explant cultures (labeled as M1–M10) were prepared from the specimens of ten patients with advanced malignancy state as described in the Materials and methods section. Prepared cultures along with cells of stabilized melanoma line Bowes and primary melanocytes HEM were used for the initial screening of their proliferation rates, total as well as free zinc content and metallothionein IIA expression. All explant melanoma cultures showed a time-dependent growth and proliferation, however, with detected inter-individual differences (for instance M5 cells versus M2 or M9 cells). Moreover, while the proliferation activity of all but one explant culture (M9) did not differ from the model malignant melanoma cell line Bowes, the growth and the reproduction rate of all studied melanoma cells significantly exceeded those of normal melanocytes HEM (Figure 1A,B). Total intracellular zinc content ranged from approximately 0.69 (M10) to 0.96 (M9) µg/mg of protein in individual explant melanoma cultures. In contrast, Bowes cells contained 0.59 and HEM cells 0.65 µg of zinc/mg of protein, respectively (Figure 1C). Unlike relatively similar total zinc levels in examined cells, free zinc pools differed significantly between individual explant melanoma cultures and control Bowes and HEM cells. The detected free zinc content ranged from the lowest levels in M5 cells (equivalent to approximately 500 light units) to the highest levels in M9 cells (equivalent to approximately 1900 light units) with reference zinc levels in Bowes cells and HEM cells being 917 and 715 light unites, respectively. (Figure 1D). The expression of metallothionein II A (MT-IIA) showed significant differences between examined cells too, with M2, M6, M7, M8, and M9 cells displaying the highest MT-IIA protein abundance whereas M1 and HEM cells lowest (Figure 1E).

### 2.2. Autophagy in Human Melanoma Cells and Melanocytes

**Figure 2:**
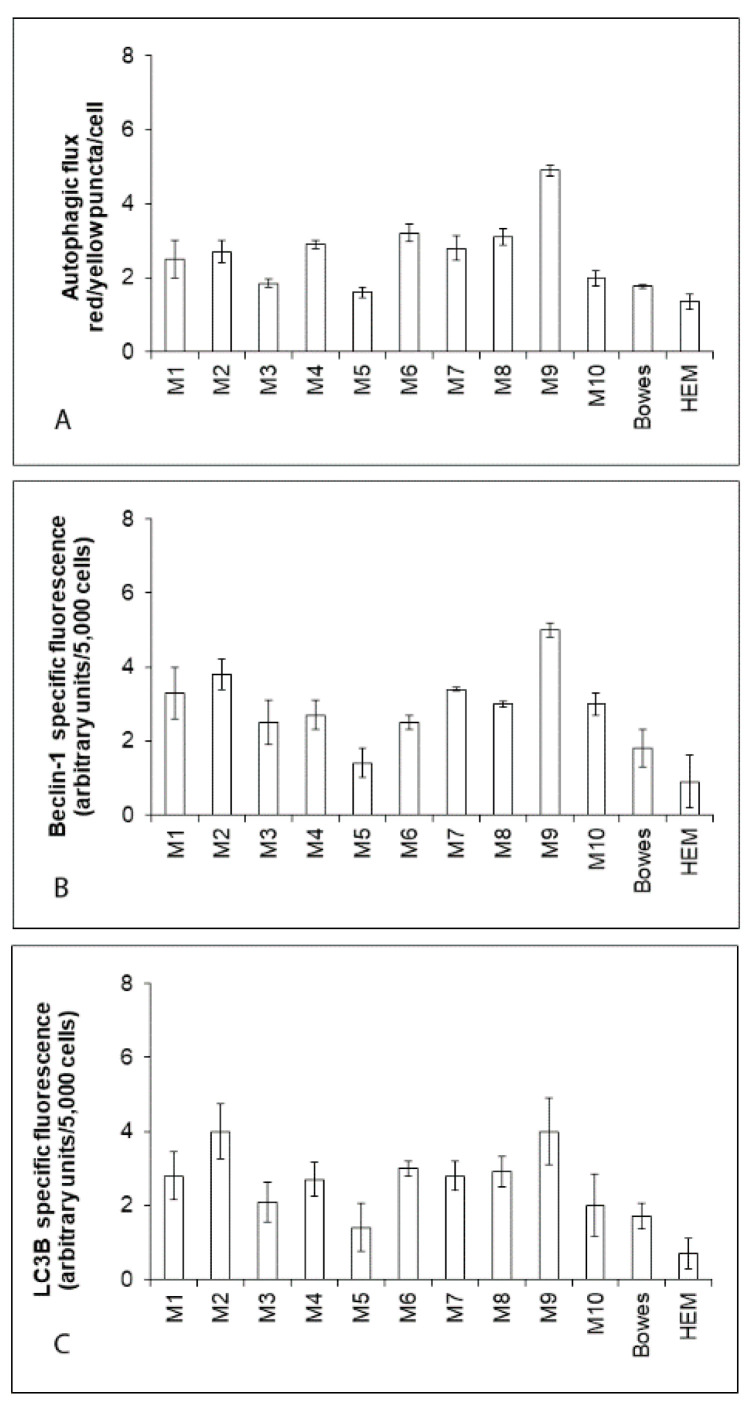
Autophagic activity in human explant melanoma cells, Bowes cell line and normal human melanocytes HEM. (**A**) Autophagic flux (the rate of formation of autophagosomes and autophagolysosomes) in individual cells was determined with help of RFP-GFP-LC3B reporter system following cell transduction and subsequent quantitation of yellow (LC3B positive autophagosomes) and red (LC3B positive autophagolysosome) fluorescence with the determined ratio of yellow/red puncta per cell. (**B**,**C**) The expression of proteins Beclin-1 and LC3B in the examined cells were determined fluorimetrically. Values represent means ± SD of at least three experiments.

In the examined cell specimens, the baseline autophagic activity was next evaluated via the autophagic flux and measured expression levels of Beclin-1 and LC3B proteins. Our obtained data show generally higher autophagic flux (i.e., the rate of formation of autophagosomes and autophagolysosomes as determined via RFP-GFP-LC3B reporter system) in explant human melanoma cultures than in Bowes and HEM cells (with the exception of M5 cells) (Figure 2A). Similarly elevated in explant melanoma cultures were Beclin-1 and LC3B expressions too (Figure 2B,C). Since the detection of autophagic flux is considered the most accurate way of measurement of changes in autophagy, this approach was used in the subsequent experiments for determination of this parameter.

### 2.3. Effects of Chronic Zinc Pyrithione Exposure on Intracellular Free Zinc and Proliferation of Bowes and HEM Cells

**Figure 3:**
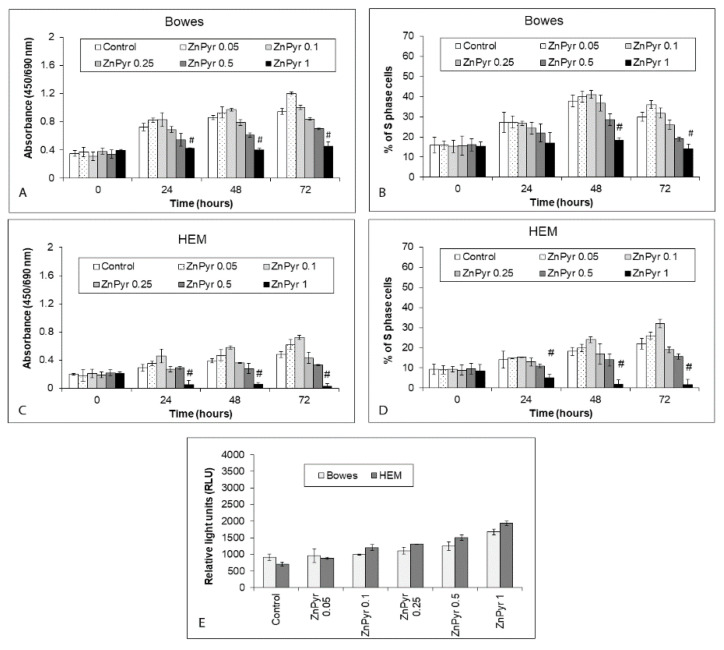
Effect of externally added zinc ionophore zinc pyrithione on the proliferation/viability and free zinc content of Bowes cell line and normal human melanocytes HEM during 72 h. Cells were exposed to zinc pyrithione in cultivation medium and (**A**–**D**) proliferation/viability was determined at individual time intervals with colorimetric WST-1 assay (measures the rate of metabolic conversion of tetrazolium salt WST-1 by mitochondrial succinate dehydrogenase in viable cells) and via measuring the proportion of S-phase cells using EdU-specific fluorescence. Values represent means ± SD of at least three experiments. # *p* < 0.05 Significantly lower than control at the same treatment interval with a one-way ANOVA test and Dunnett’s post-test for multiple comparisons. Free (labile) zinc content of treated cells (**E**) at 48 h was determined using fluorimetry of the zinc-specific dye Newport Green diacetate. Results represent means ± SD of five experiments.

To determine an optimal working concentration of zinc ionophore to be used for zinc loading experiments in our model, differing concentrations of zinc pyrithione were initially tested in Bowes and HEM control cells. As shown in Figure 3A–D, lower zinc pyrithione concentrations (up to 0.1 µM) had mildly inducing effects on proliferation of both Bowes and HEM cells upon a corresponding elevation in free intracellular zinc content. Conversely, higher zinc pyrithione concentrations while still increasing intracellular free zinc levels tended to inhibit cell viability and proliferation mildly (0.25 to 0.5 µM) and significantly (1 µM). In particular, zinc pyrithione concentration of 0.5 µM did not induce any significant cytotoxicity in both Bowes and HEM cells despite their elevated intracellular free zinc levels. Still, its suppressive effects were relatively more extensive in Bowes cells compared to HEM cells (Figure 3A–D), suggesting possible similar sensitivity of malignant cells towards this concentration. This concentration was thus chosen for further experiments with select explant melanoma cultures.

### 2.4. Effects of Chronic Zinc Pyrithione Exposure on Intracellular Free Zinc and Viability/Proliferation of Explant Melanoma Cultures

**Figure 4:**
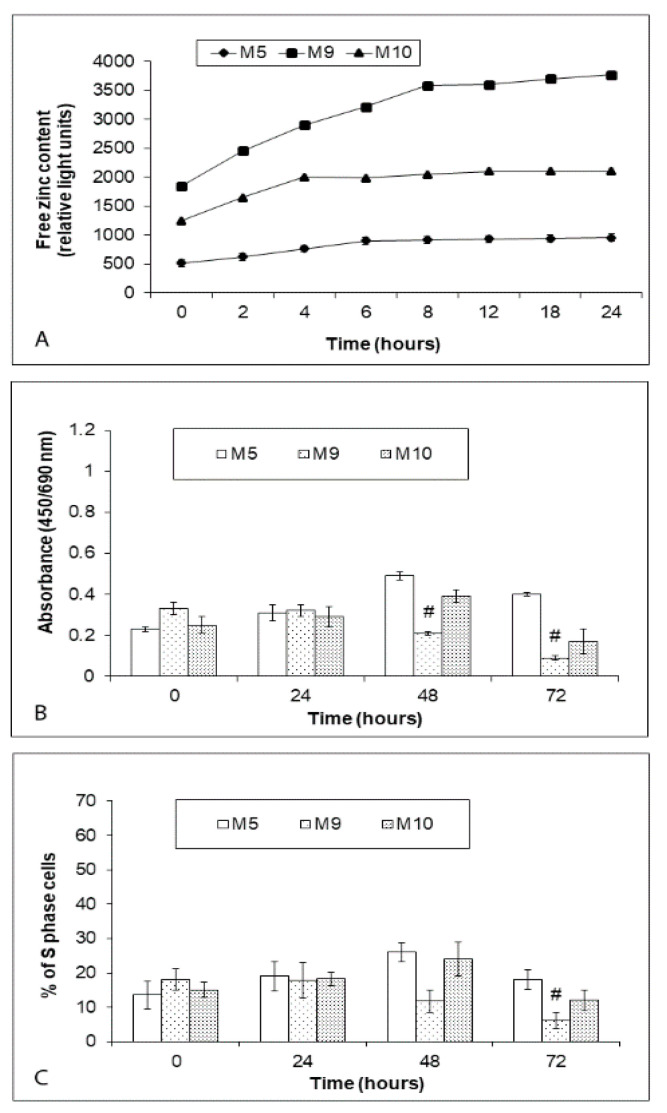
Effect of external 0.5 μM zinc pyrithione on accumulation of intracellular free zinc (**A**) and proliferation/viability (**B**,**C**) of explant human melanoma cultures with lower free zinc stores (M5), average free zinc stores (M10) and higher free zinc stores (M9) during 24 h (free zinc content) to 72 h (proliferation/viability). Cells were exposed to zinc pyrithione and intracellular free zinc levels were monitored during 24 h using fluorimetry of the zinc-specific dye Newport Green diacetate. Proliferation/viability of cells exposed to zinc pyrithione was estimated with colorimetric WST-1 assay (determines the rate of metabolic conversion of tetrazolium salt WST-1 by mitochondrial succinate dehydrogenase in viable cells) and via measuring the proportion of S-phase cells using EdU-specific fluorescence. Data represent the mean ± S.D. of three independent experiments. # *p* < 0.05 Significantly lower than the beginning of treatment with the same concentration with a one-way ANOVA test and Dunnett’s post-test for multiple comparisons.

For the following experiments with zinc, 3 melanoma explant cultures were selected out of 10 obtained to represent determined heterogeneity in cell proliferation, zinc content, and autophagic rate. Among them were M5 cells (the least proliferation dynamics over 72 h and the lowest free zinc content as well as autophagy), M9 cells (the highest proliferation rate over 72 h and the highest free zinc content and autophagy flux), and M10 cells (an average proliferation dynamics over 72 h and the free zinc content and autophagy rate comparable with the remaining explant cultures). When exposed to 0.5 µM zinc pyrithione over 24 h, free zinc pools grew in all three treated explant cultures but with a differing dynamics and with a distinct final reached level. Specifically, in M5 cells a gradual increase in free zinc content occurred during first 6 h of exposure with a very little change detected until 24 h. A similar trend was noted in M10 cells, however, the final free zinc levels at the end of the experiment nearly doubled unlike in M5 cells. In M9 cells, a steep intracellular free zinc accumulation occurred during the first 10 h of exposure, then the growth stabilized and continued very slightly until 24 h. At this time interval free zinc pools more than doubled too (Figure 4A).

While the mentioned changes in free zinc levels produced no change in proliferation rate/viability of all three explant cultures during 24 h, a significant drop in proliferation/viability occurred at 48 h in M9 cells but not in M5 and M10 cells and it became even more pronounced at 72 h. At this time interval, proliferation activity/viability remained relatively high in M5 cells while it decreased in M10 cells and very markedly in M9 cells (Figure 4B).

### 2.5. Increases in Intracellular Free Zinc Associate with Varying Levels and Dynamics of Autophagy in Melanoma Explant Cultures

**Figure 5:**
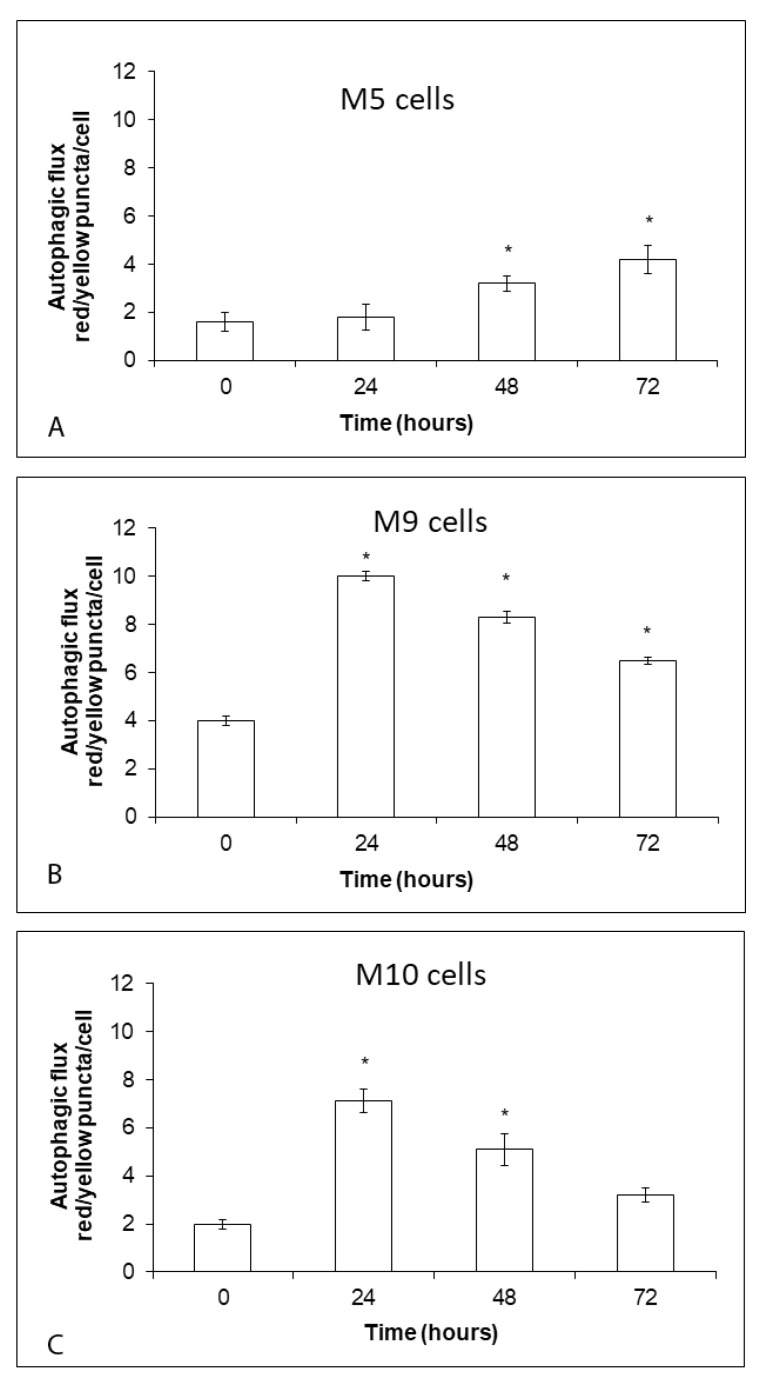
Effect of external 0.5 μM zinc pyrithione on autophagy flux in explant human melanoma cultures with lower free zinc stores (**A**), average free zinc stores (**B**) and higher free zinc stores (**C**) during 72 h. Cells of melanoma cultures transduced with RFP-GFP-LC3B reporter system were exposed to zinc pyrithione and at regular time intervals their autophagy flux was measured by quantization of yellow (LC3B positive autophagosomes) and red (LC3B positive autophagolysosome) fluorescence and determination of the ratio of yellow/red puncta per cell. Data represent the mean ± S.D. of three independent experiments. * *p* < 0.05. Significantly higher than the beginning of treatment with the same concentration with a one-way ANOVA test and Dunnett’s post-test for multiple comparisons.

We measured autophagy flux in the studied explant melanoma cultures exposed to 0.5 µM zinc pyrithione during 72 using RFP-GFP-LC3B reporter system. In M5 cells, which presented the lowest basal autophagy, its activity grew in a time-dependent measure and reached its maximum at 72 h (Figure 5A). On the other hand, autophagy in M9 cells (the highest basal level) steeply grew during first 24 h and then gradually but slightly declined (Figure 5B). This trend was recorded in M10 cells too, however, a decrease in autophagic activity was more significant and at 72 h this activity reached almost basic levels (Figure 5C).

### 2.6. Accumulation of Intracellular Zinc in Melanoma Explant Cultures Leads to Morphologically and Biochemically Distinct Cell Damage/Death Mode

**Figure 6:**
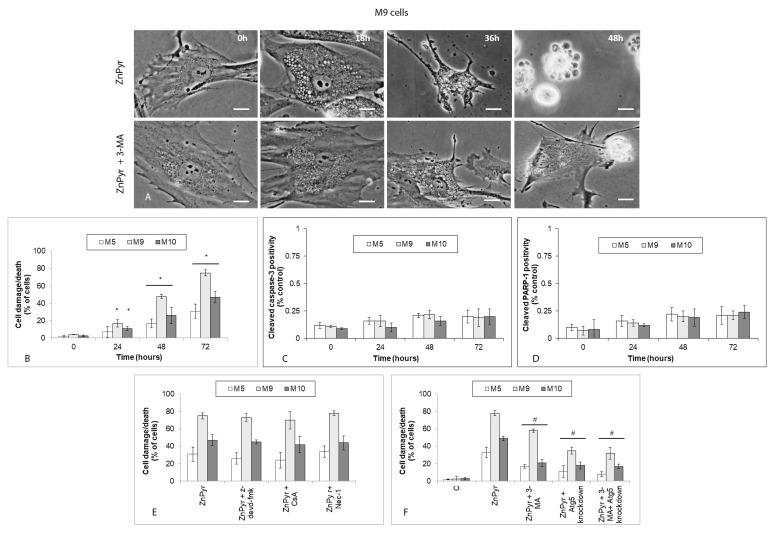
Cell damage/death of explant human melanoma cultures with lower free zinc stores (M5), average free zinc stores (M10) and higher free zinc stores (M9) exposed to 0.5 μM zinc pyrithione (ZnPyr) during 72 h. Cells (with normal or downregulated expression of Atg5) were treated with zinc pyrithione alone or with pancaspase inhibitor z-devd-fmk, cytochrome c mitochondrial release blocking cyclosporine A, necroptosis inhibitor necrostatin 1 (Nec-1) or autophagy inhibiting 3-methyladenine (3-MA) and their effect on cell damage/death was evaluated by morphometric analysis of cell microgragraphs obtained from time-lapse microscopy (**A**,**B**,**E**,**F**) or fluorimetric evaluation of active caspase-3 and cleaved PARP-1 presence in the cells (**C**,**D**). Results represent means ± SD of five experiments. (**A**) Phase contrast microscopy, 600×, bar 1 µm. (**B**) * *p* < 0.05 significantly higher compared to the beginning of treatment, (**F**) # *p* < 0.05 significantly lower than zinc pyrithione only treated cells at the same time interval with one-way ANOVA test and Dunnett’s post-test for multiple comparisons.

Since the treatment of explant melanoma cultures with 0.5 µM zinc pyrithione resulted in the suppressed proliferation and decreased viability, we sought to check the morphology of these cells using time-lapse phase contrast microscopy. Image analysis of thus acquired micrographs revealed in the studied populations the presence of heavily vacuolated cells and cells in a varying degree of destruction in the absence of classical hallmarks of apoptosis. Typically, thus affected cells were collapsed with curiously spiked peripheries devoid of cytoplasm, sometimes at later treatment intervals with features of secondary necrosis (Figure 6A). Quantitatively, the proportion of damaged/dying cells grew linearly in all three melanoma explant cultures until 72 h but again it differed in its extent between particular cells. The highest determined cell damage/death rate occurred with M9 cells (75%) and the lowest one in M5 cells (30%). Also, in both M9 and M10 cells cell damage/death reached a significant level already at 24 h of treatment unlike in M5 cells (Figure 6B).

An independent analysis of the involvement of active caspase-3 and cleaved PARP-1—i.e., the typical effector and substrate of apoptosis—in 0.5 µM zinc pyrithione induced cell damage/death did not find their significant presence in the studied models at all treatment intervals (Figure 6C,D). Furthermore, pretreatment of cells with pan-caspase inhibitor or with cyclosporine A—a specific inhibitor of cytochrome c—had no effect of cell damage/death rate. Similar result was observed in case of the employed inhibitor of necroptosis. On the other hand, both autophagy inhibitor 3-MA as well as targeted downregulation of autophagy gene Atg5 significantly reduced zinc pyrithione-dependent cell damage/death (Figure 6A,E,F).

### 2.7. Intracellular Zinc Stimulates Lysosomal Activity and Time-Dependent Damage of Lysosomal Membrane

**Figure 7:**
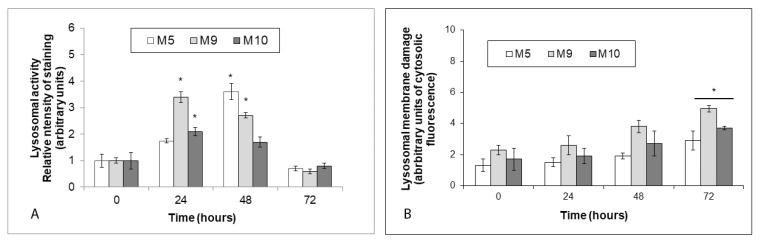
Lysosomal activity and lysosomal membrane damage in explant human melanoma cultures with lower free zinc stores (M5), average free zinc stores (M10) and higher free zinc stores (M9) exposed to 0.5 μM zinc pyrithione during 72 h. Cells were exposed to external zinc pyrithione and (**A**) fluorescence emission of proteolyzed DQ-Green BSA reflecting lysosomal activity and (**B**) acridine orange diffuse green cytoplasmic fluorescence indicative of lysosomal membrane damage were determined fluorimetrically. Results represent means ± SD of at least three independent experiments. * *p* < 0.05 Significantly higher compared to the beginning of treatment with one-way ANOVA test and Dunnett’s post-test for multiple comparisons.

Lysosomal activity in treated melanoma explant cultures grew time-dependently, with its maxima being reached at 24 h (M9 and M10 cells) and 48 h (M5 cells), respectively. Still, at the end of the experiment this activity dropped below the basal levels (Figure 7A). Integrity of lysosomal membrane, on the other hand, gradually decreased in time in all treated cells, and reached a significant extent with M9 and M10 cells at 72 h of exposure. Conversely, lysosomal membrane damage in M5 cells while elevated was not significant during the entire course of treatment (Figure 7B).

### 2.8. Early Changes in Mitochondrial Functions and Mitophagy in Zinc Pyrithione Exposed Cells

**Figure 8:**
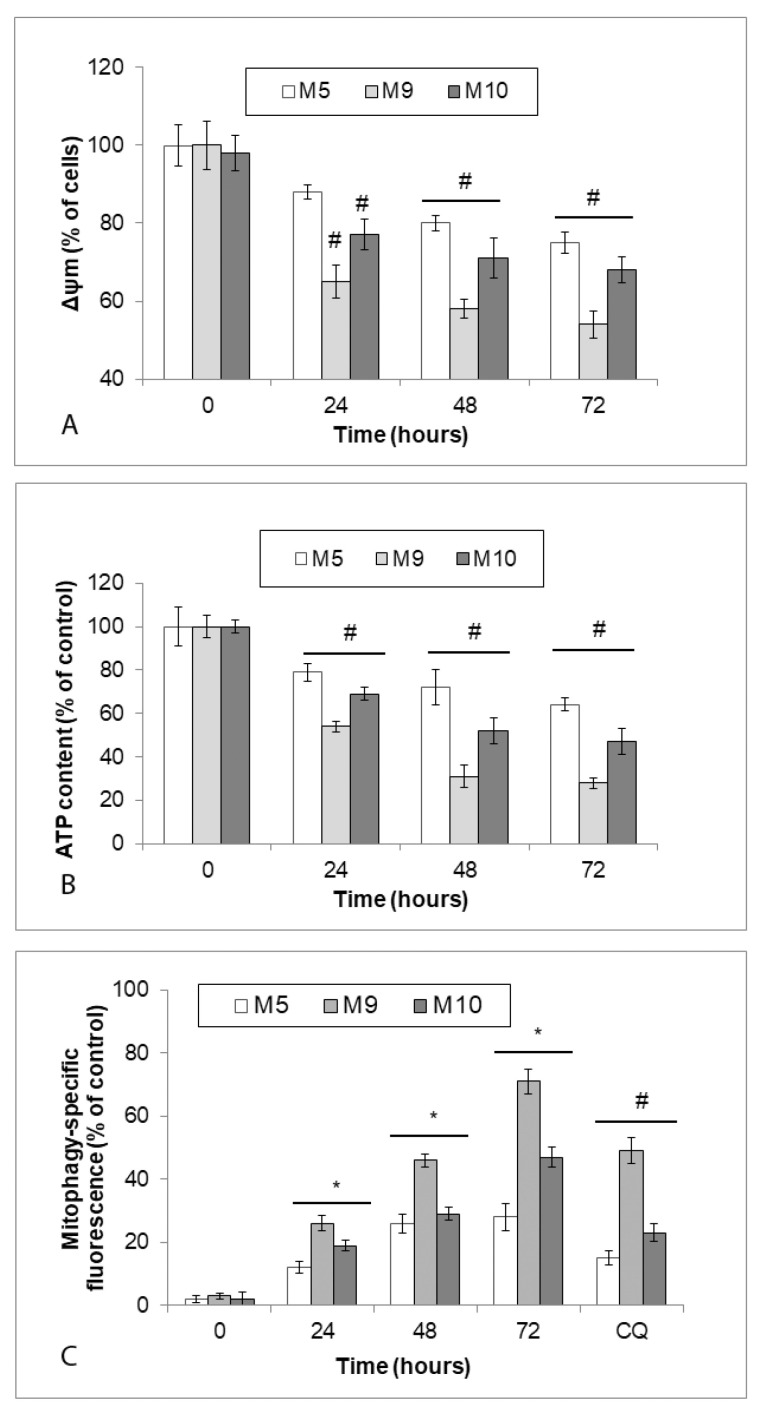
Mitochondrial membrane potential (Δψm) changes, ATP production and mitophagy in explant human melanoma cultures with lower free zinc stores (M5), average free zinc stores (M10) and higher free zinc stores (M9) exposed to 0.5 μM zinc pyrithione during 72 h. Cells were exposed to external zinc pyrithione and (**A**) loss of Δψm measured by decreased red fluorescence of JC-1 was determined in at least 1000 cells visualized by fluorescence microscopy. Results represent means ± SD of at least three independent experiments. # *p* < 0.05 significantly lower compared to the beginning of treatment with one-way ANOVA test and Dunnett’s post-test for multiple comparisons. (**B**) ATP production was measured in cell lysates by ATP bioluminescent assay kit (**C**). Mitophagy-specific fluorescence (Mitophagy Detection Kit) in cells exposed to zinc pyrithione alone or together with autophagy inhibitor chloroquine was determined fluorimetrically. Results represent means ± SD of at least three independent experiments. * *p* < 0.05 significantly higher compared to the beginning of treatment, # *p* < 0.05 significantly lower compared to the beginning of treatment with one-way ANOVA test and Dunnett’s post-test for multiple comparisons.

Concerning the relevance of mitochondria as targets of intracellular zinc, their status and activity in the zinc pyrithione-treated melanoma cells were further evaluated. Results of preliminary experiments with specific mitochondrial tracer dye Mitotracker revealed a significant loss in mitochondrial mass of treated melanoma cells (data not shown). Mitochondrial activity (as measured by mitochondrial membrane potential—Δψm changes) steeply declined during first 24 h of exposure to 0.5 µM zinc pyrithione (M9 and M10 cells) whence upon it continued to decrease more slowly until the end of the treatment (72 h). Significant differences between particular explant cultures were noted (Figure 8A). The same trend was observed in case of ATP production (Figure 8B). Conversely, in zinc-loaded cells, mitophagy specific fluorescence increased, suggesting an increased loss of mitochondria in treated cells which was further proved by using inhibitor chloroquine (CQ) (Figure 8C).

### 2.9. Intracellular Zinc Enhances Production of Superoxide in Melanoma Explant Cultures

**Figure 9:**
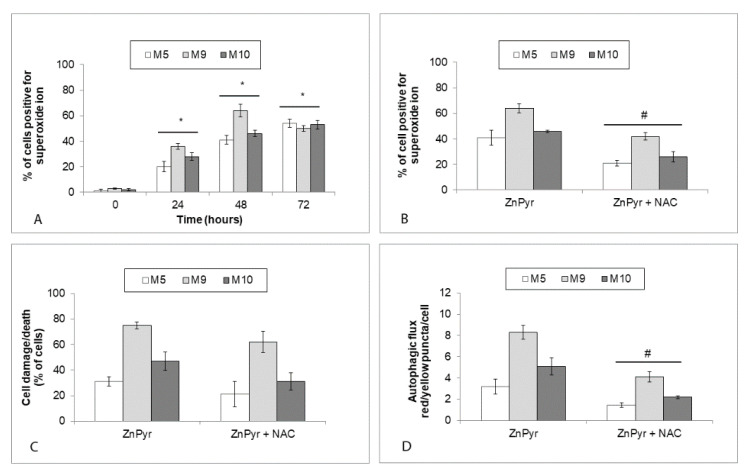
Generation of superoxide in explant human melanoma cultures with lower free zinc stores (M5), average free zinc stores (M10), and higher free zinc stores (M9) exposed to 0.5 μM zinc pyrithione (ZnPyr) during 72 h. Cells were exposed to external zinc pyrithione and generation of superoxide (**A**) was measured in individual cells via fluorimetric measurement of MitoSOX™ Red positivity. Results represent means ± SD of at least three independent experiments. * *p* < 0.05 significantly higher compared to the beginning of treatment with one-way ANOVA test and Dunnett’s post-test for multiple comparisons. Effect of pretretement of cells with antioxidant *N*-acetyl cysteine (NAC) on (**B**) generation of superoxide, (**C**) cell damage/death, and (**D**) autophagic flux at 48 h. Cell damage/death was evaluated morphometrically in cell micrographs obtained with time-lapse microscopy and autophagic flux was determined by quantization of of yellow (LC3B positive autophagosomes) and red (LC3B positive autophagolysosome) fluorescence with the determined ratio of yellow/red puncta per cell. Results represent means ± SD of at least three independent experiments. # *p* < 0.05 significantly lower compared to the ZnPyr treated cells only with one-way ANOVA test and Dunnett’s post-test for multiple comparisons.

Measurement of superoxide production in zinc pyrithione-treated cells showed its markedly increased levels in exposed cells already at 24 h and their continued elevated presence throughout the experiment (Figure 9A). The employed antioxidant NAC reduced superoxide levels in zinc-loaded cells as well as the rate of autophagic flux although its effect on cell damage/cell death was marginal (Figure 9B–D).

## 3. Discussion

Skin cells are rich in zinc but whether this element plays any specific role in the development and progression of melanoma is until today unclear. Current evidence (scarce and mostly indirect) suggests an impaired zinc content in melanoma patients [29,30,31] as well as its specific localization within the tumor [32] but nothing is known about the content and sensitivity to zinc of melanoma cells in advanced stages of this malignancy.

In this study, we used melanoma explant cultures established from samples of 10 patients with advanced melanoma and compared them with stabilized Bowes cell line and normal human melanocytes HEM. Initial analyses confirmed heterogeneous proliferation activities as well as zinc content (in particular free zinc) in individual explant cultures which were in these parameters roughly similar to Bowes cell line but mostly differed from HEM cells. Also, the present autophagic activity as reflected by the rate of autophagic flux as well as the expression of Beclin-1 and LC3B varied widely among explant cultures but its levels were always higher than in Bowes cells (with exception of M5 cells) and HEM cells. These last findings thus seem to confirm earlier observations that autophagy is enhanced in advanced stages of melanoma [17] while bringing interesting new facts about uneven but often higher free zinc content in particular melanoma cells. Moreover, we also evidenced that the expression of metallothionein IIA, which regulates intracellular free zinc concentrations, did not always specifically correlate with detected free zinc levels in individual explant cultures. This finding stresses the fact that intracellular zinc management in advanced melanoma cells might not be specifically dependent on metallothionein IIA only and likely includes other zinc-specific regulators and mechanisms whose nature awaits future detailed analyses. These should, among other objectives, confirm whether varying zinc content, proliferation, and autophagy rates specifically interrelate and represent a universal feature of advanced malignant melanoma cells with potentially new and useful biological significance.

Since melanoma cells harbored varied free zinc content, we focused next on biological effects of further zinc loading in those cultures with previously determined low free zinc content (M5 cells), average free zinc content (M10 cells), and high free zinc content (M9 cells). For zinc loading experiments zinc pyrithione acting as a zinc ionophore has been used at concentration 0.5 µM which we determined to be nontoxic in Bowes and HEM cells. Our results showed that all three melanoma cultures exposed to zinc pyrithione increased their free zinc content but with individual timing and the final level attained. Moreover, such zinc loading had a final negative effect on their viability and proliferation in the presence of markedly elevated autophagic flux proportional to the intracellular free zinc content. To this extent, several recently published reports evidenced the fact that zinc has positive effects on autophagy [33,34]. Specifically, autophagy rates have been proposed to correlate with free zinc levels in cells as for instance shown in experiments with tamoxifen in MCF-7 cell line where authors demonstrated accumulation of free zinc in autophagosomes and lysosomes [35]. The exact mechanism(s) whereby zinc influences autophagy are not elucidated; however, several lines of evidence indicate the possible role of metallothioneins [36], extracellular-signal-regulated kinases, or select microRNAs [37].

Besides enhanced autophagy and decreased proliferation/viability, an excessive accumulation of free zinc in the studied explant melanoma cultures led to their observable cell damage/death whose rates increased in a time-dependent manner. Morphologically, cells developed extensive intracellular vacuolization and underwent gradual collapse bearing characteristics of neither classical apoptosis nor necrosis (Figure 6A). This result was further confirmed by the absence of some established apoptotic markers (for instance active caspase-3 and cleaved PARP-1), preserved integrity of cell membrane (data not shown) and the lack of effect of several used apoptotic and necroptotic pharmacological inhibitors. Conversely, autophagosome/autolysosome inhibitor 3-MA as well as downregulation of Atg5 protein exhibited a marked suppressive effect on zinc pyrithione-induced cell damage/death, suggesting that free zinc-induced autophagy might be directly responsible for resulting cell damage/death with such a scenario corresponding to the defined autophagy-dependent cell death [38]. To date, this cell death modality has been described in a limited number of cases including enforced expression of *H-Ras* oncogene in ovarian cancer cells [39] or in A549 lung carcinoma cells treated with resveratrol [40]. In both instances, individual autophagy-regulating genes such as *ATG5, ATG7*, or *ULK1* were found to be directly involved in the induced process of cell death. Concerning the efficient suppression of zinc-induced damage by downregulation of Atg5 protein in exposed melanoma cells, the active role of this gene is clearly confirmed in the present model. However, in cells free zinc may interact with many other targets including zinc buffering metallothioneins [41] and zinc sequestering endoplasmic reticulum, Golgi body, mitochondria, lysosomes, or other subcellular compartments [42,43,44] whose role in zinc-induced autophagy/damage/death has been acknowledged [37]. The extent of their involvement in advanced melanoma is currently not known although their possible participation may not be ruled out.

Zinc may accumulate in lysosomes, contribute to the changes in lysosomal luminal acidity and when rapidly released it stimulates autophagy and cell injury [45]. Although we have not directly measured lysosomal zinc content in our experimental setting, our current findings show an increase in lysosomal activity in a time frame correlating with maximum free zinc accumulation in the cells. Such a finding seems to indicate that either free zinc did not accumulate in lysosomes to the extent necessary to negatively affect lysosomal performance and final autophagic efficiency or may mean that zinc did enter into the lysosomes, which nevertheless preserved their activity along with the overall autophagy. While we have not analyzed further in detail this aspect of zinc biology, we have discovered that mitochondria, another important target of intracelullar free zinc, demonstrated significant changes in their mass and activity as seen by rapidly declining mitochondrial membrane potential, ATP production, and growth in superoxide levels. In particular, oxidative stress generated by damaged mitochondria then may have led to their increased recycling via mitophagic activity of melanoma cells and contributed to autophagy-mediated cell damage and death. Oxidative stress is also known to induce lysosomal membrane permeabilization (LMP) contributing in many cases to the ultimate cell damage and cell death [46]. Our present data demonstrate significant lysosomal content leakage in treated melanoma cells, however, at 72 h of treatment only, i.e., when the cells were already extensively damaged (Figure 7B). It is thus possible that in our model LMP occurred gradually which allowed the cell to compensate for incurred lysosomal membrane damage for some time. Alternatively, LMP could have been somehow masked by enhanced involvement of lysosomal compartment in autophagy. The inhibitory effect of NAC on LMP which proved to be low (data not shown) further confirms that zinc pyrithione induced generation of the superoxide had a relatively limited effect on lysosomal membrane damage in the present model. In fact, next to oxidative stress other contributing elements must have been involved in the final cell damage and cell death too as demonstrated by the lack of significant protection of treated cells by antioxidant NAC.

Several limitations of this study must be acknowledged. Firstly, it is the number of analyzed melanoma samples, in particular with respect to the acknowledged melanoma heterogeneity and thus robustness of acquired data. Consequently, our identified melanoma cell populations with low/medium/high free zinc content may well point at either a potentially novel general hallmark in melanoma progression or just represent concrete specific (perhaps extreme) tumor phenotypes related to the particular melanoma case only. This fact is certainly relevant since our analyses of biological responses of these cells to zinc pyrithione revealed significant differences in their autophagy, proliferation, and death rates. Their satisfactory explanation would require a detailed comparison between all known intracellular zinc regulating factors including zinc transporters, buffers, and mufflers as well as a general profiling of other genotypic and phenotypic characteristics of these cells, ideally from large sample sets. Still, given the rather extensive involvement of zinc in various cells’ activities, it would be no simple task, with a very likely existence of interrelated, not easily separable mechanisms of a hitherto undefined spatiotemporal nature.

Accordingly, it must also be highlighted that acutely increased intracellular zinc may not produce consistent and predictable suppressive effects towards melanoma cells growth and proliferation, thereby necessitating caution when analyzing and interpreting thus acquired results. With an absence of any gold standard for defining changes in intracellular zinc management in melanoma, additional approaches to refining this aspect of melanoma biology might include molecular analyses of expression and activity of relevant zinc-related markers at diverse stages of melanoma development as well as matched clinical data about overall zinc status and intake.

Taken together, we report here that established melanoma explant cell cultures representing advanced form of this malignancy show a considerable heterogeneity in their free zinc content, proliferation, as well as autophagic flux which are generally similar to those in stabilized cell line Bowes but higher that in normal human melanocytes HEM. The use of zinc pyrithione at the concentration of 0.5 µM rapidly increases free zinc levels as well as autophagy in melanoma cells regardless of their original free zinc content. Such a treatment also suppresses proliferation of these cells and induces their damage/death in a cell type specific manner. This death is neither apoptosis not necrosis and its phenotype corresponds most likely to autophagy-dependent cell death. Mechanistically, excess free zinc in melanoma cells damages their mitochondria which are recycled via lysosomes and mitophagy with concurrently elevated superoxide species. Both autophagy as well as reactive oxygen species and other as yet undefined elements contribute to the final injury and cell death. Thus present results show for the first time that acutely increased intracellular free zinc in melanoma cells specifically enhances their autophagic activity via mitochondria and lysosomes which leads to autophagic cell death. While biologically relevant, this discovery may contribute to our understanding and exploration of zinc in relation to autophagy as a means of controlling melanoma growth and survival.

## 4. Materials and Methods

### 4.1. Explant Melanoma Cultures

Tumor specimens were acquired from 10 patients (5 males and 5 females, age 60–72 years, the stage according to Clark IV) undergoing surgery due to a diagnosis of malignant melanoma of a varying stage at University Hospital in Hradec Kralove. The study was approved by the local ethical committee and patients gave their written consent. Samples were homogenized by cutting and pressing through a colander and obtained cell suspension was assorted via gradient centrifugation (1008 RCF, 20 min, 25 °C). Assorted suspensions were maintained in growth medium containing 100 µg/mL geneticin (5 days) to remove contaminating keratinocytes and fibroblasts. Extant melanoma cells were collected, washed, and isolated with magnetic separation. Viable cells (as determined by Trypan blue exclusion assay) were then seeded into cultivation flasks and kept in RPMI-1640 medium with 1% penicillin/streptomycin, 15% FBS, humulin N 100 IU/mL, transferrin 2 mg/mL and melanocyte-growth supplement HMGS (Thermo Fisher Scientific, Prague, Czech Republic) upon standard conditions (37 °C, 5% CO_2_) in an incubator. Cultures were passaged using 0.05% trypsin/EDTA upon reaching 90% confluence. Identity of melanoma cells was confirmed using S-100 and anti-cytokeratin antibodies. Thus, characterized cells were in the subsequent experiments labeled as M1 to M10 samples.

### 4.2. Cell Lines

Melanoma cell line Bowes (ATCC, No. CRL–9607, Manassas, VA, USA) was maintained in DMEM (Sigma-Aldrich, St. Louis, MO, USA) with 10% fetal bovine serum (Gibco, Prague, Czech Republic), 100 U/mL penicillin, and 100 µg/mL streptomycin. Normal human melanocytes HEM (Cell Applications, Inc., San Diego, CA, USA) were cultured in full HEM growth medium (Cell Applications, Inc., San Diego, CA, USA). Cultures were kept in an incubator at 37 °C and 5% CO_2_ atmosphere and were passaged two times a week using 0.05% trypsin/EDTA upon reaching 90% confluence. Only mycoplasma-free cells were used for the experiments.

### 4.3. Chemicals

JC-1, Newport Green™ DCF diacetate, DQ-Green BSA and Click-iT EdU Kit were acquired from Molecular Probes, Inc. (Eugene, OR, USA). Zinc pyrithione, acridine orange, cyclosporin A, monodansylcadaverine (MDC), *N*-acetyl cysteine (NAC), 3-methyladenine (3-MA), chloroquine (CQ), 3-[(3-cholamidopropyl)dimethylammonio]-1-propanesulfonic acid (CHAPS), horseradish peroxidase, Triton-X, dithiotreitol (DTT), bisBenzimide H 33342 trihydrochloride (Hoechst 33342) and 4’,6-Diamidino-2-Phenylindole (DAPI) were obtained from Sigma-Aldrich (St. Louis, MO, USA). WST-1 was purchased from Roche Diagnostics (Manheim, Germany). Caspase-3 inhibitor z-devd-fmk was from ICN Biomedicals Inc. (Irvine, CA, USA). Necrostatin-1 was from Santa Cruz Biotechnology, Inc. (Dallas, TX, USA). Primary and secondary antibodies were from Cell Signaling Technology (Danvers, MA, USA). All other chemicals were of the highest analytical grade.

### 4.4. Treatment Conditions

Zinc pyrithione was dissolved in a serum-free medium and stored until use as a stock solution of 1 mM in a refrigerator (4 °C). Inhibitors and other modulators were used in the following way: z-devd-fmk (caspase-3 inhibitor, 5 µM—added to cells simultaneously with zinc pyrithione), cyclosporin A (5 µM—supplemented to cells 30 min before exposure to zinc pyrithione), 3-MA—autophagy inhibitor (5 mM—supplemented to cells 30 min prior to exposure to zinc pyrithione), Chloroquine—autophagy inhibitor (100 µM—added to cells 30 min prior to exposure to zinc pyrithione), Necrostatin-1 (60 µM—supplemented to cells for 1 h during exposure to zinc pyrithione), NAC—*N*-acetyl cysteine (200 µM—added to cells 24 h prior to zinc pyrithione treatment).

### 4.5. Proliferation and Viability Assay

Explant human melanoma cultures, human melanoma cell line Bowes, and normal human melanocytes HEM in cultivation medium with 10% FBS were seeded in 96-well microtiter plates (Nunclon, Roskilde, Denmark) at the initial seeding density of 6000 cells/well with the first column of wells without cells (blank). At each time interval of measurement, 100 µL of WST-1 was added to each well. The cells were further incubated for 2 h in an incubator. Thereafter, the absorbance was measured using the multiplate reader TECAN SpectraFluor Plus (TECAN Austria GmbH, Grödig, Austria) at 450 (excitation) and 690 nm (emission).

### 4.6. S-Phase Cell Fraction Assay

Explant human melanoma cultures, human melanoma cell line Bowes and normal human melanocytes HEM were seeded in 96-well microtiter plates (Nunclon, Roskilde, Denmark) at the initial seeding density of 6000 cells/well. At individual time intervals, cells were washed with PBS and the medium with EdU (10 µM) was added to each well for 2 h. Then, medium was removed, cells were washed with PBS and fixed with paraformaldehyde (15 min) with subsequent permeabilization (0.5% Triton-X, 20 min, 25 °C). Following the next washing cycle with PBS, EdU buffer additive was added to cells (30 min, dark, 25 °C). Finally, thus treated cells were washed again with PBS, postlabeled with DAPI and their specific S-phase fluorescence visualized, recorded and analyzed by Cell scoring module of MetaXpress^®^ Image Acquisition and Analysis Software.

### 4.7. Intracellular Zinc Concentrations

Total and free intracellular zinc content in the explant human melanoma cultures, human melanoma cell line Bowes, and normal human melanocytes HEM were determined as described before [47]. The trypsinized and rinsed cells were dissolved in 0.35 mL 0.8% nitric acid and assayed for zinc with the inductively coupled plasma emission spectrometer MSD 5972 (Agilent Technologies, Waldbronn, Germany). Prior to analysis, aliquots of the cell samples were assayed for protein content using a BCA assay (Bicinchoninic acid kit for protein determination, Sigma-Aldrich, Prague, Czech Republic). Changes in total intracellular zinc content were expressed as µg of zinc/mg of protein.

Free intracellular zinc levels in the assayed cells were determined fluorimetrically. The cells grown in black-bottom 96-well plates were incubated with Newport Green diacetate (5 μmol in PBS, dark, 30 min at 37 °C). Fluorescence intensity was determined by the multiplate reader TECAN SpectraFluor Plus (TECAN Austria GmbH, Grödig, Austria). The results in relative light units were obtained from the raw data minus reagent blank, with changes expressed as a percentage of controls.

### 4.8. Autophagy

Baseline autophagy levels in explant human melanoma cultures, human melanoma cell line Bowes, and normal human melanocytes HEM were determined via (1) measurement of autophagic flux (the rate of autophagosome and autophagolysosome formation) and (2) Beclin-1 and LC3B expression.

Autophagy flux (the formation of autophagosomes and autophagolysomes) in control and zinc pyrithione treated cells was detected using the Premo™ Autophagy Tandem Sensor RFP-GFP-LC3B Kit (Thermo Fischer Scientific, New York, NY, USA) which enables discrimination between acidic and neutral LC3B-positive vesicles. Based on the manufacturer’s instructions, melanoma cells grown on coverslips were transduced with 10 μL of BacMam reagents containing the RFP-GFP-LC3B and cultured for 24 h. Following the treatment with zinc pyrithione, cells were rinsed in PBS, nuclei were stained with Hoechst 33342 mounted into VectaCell Trolox Antifade Reagent (Vector Laboratories, Inc., Burlingame, CA, USA). Fluorescent images were obtained with Nikon Eclipse N*i* microscope (Nikon, Prague, Czech Republic). Numbers of yellow (LC3B positive autophagosomes) and red (LC3B positive autophagolysosome) puncta per individual cell were determined manually using the multidimensional analysis module of NIS Elements AR software. Autophagic flux was expressed as the ratio of red to yellow puncta per cell. In total, 250 cells per coverslip were analyzed. Samples were done in triplicates.

For Beclin-1 and LC3B expression detection, cells in 96-well plates with black bottom were fixed in 4% paraformaldehyde and permeabilized with cold methanol/Triton-X in 5% BSA. Then they were incubated with the following primary antibodies: anti-Beclin-1 1:100 and anti-LC3B (1:150) at 4 °C for 1 h. After washing with cold PBS (5 min, 25 °C), FITC-conjugated goat anti-rabbit secondary antibody was supplied for 1 h at 4 °C. Samples were then rinsed in PBS, counterstained with DAPI and analyzed by Cell scoring module of MetaXpress^®^ Image Acquisition and Analysis Software. Obtained data are expressed as relative intensity of Beclin-1 and LC3B-specific fluorescence in arbitrary units per 5000 cells.

### 4.9. Mitochondrial Transmembrane Potential (Δψm)

Control and zinc pyrithione-treated cells of explant human melanoma cultures grown in cytospin chambers were rinsed in warm medium and stained with cationic JC-1 dye (10 μg/mL, 15 min, 37 °C). Changes in Δψm in at least 1000 cells were assessed under the fluorescence microscope Nikon Eclipse E 400 (Nikon, Prague, Czech Republic). Photographs were taken using the software NIS Elements AR 3.20 and the numbers of cells with unchanged and decreased Δψm in each time interval were determined. Results were expressed as percentage of cells with given Δψm.

### 4.10. Superoxide Production

Control and zinc pyrithione-treated cells of explant human melanoma cultures grown in 96-well plates with black bottom were incubated with MitoSOX™ Red solution (5 µM, 20 min, 37 °C), rinsed in warm medium and the specific fluorescence reflecting superoxide was analyzed by Cell scoring module of MetaXpress^®^ Image Acquisition and Analysis Software. Results were expressed as percentage of cells positive for superoxide ion.

### 4.11. ATP Production

ATP content in lysates of explant human melanoma cultures (untreated or treated with zinc pyrithione) was measured by ATP bioluminescent assay kit (Sigma-Aldrich, cat. no. FLAA, Prague, Czech Republic) as recommended by manufacturer. Results were expressed as a percentage of control.

### 4.12. Mitophagy

Control and zinc pyrithione-treated cells of explant human melanoma grown in 96-well plates with black bottom were washed with PBS and incubated in 100 nM Mitophagy Dye Solution (Mitophagy Detection Kit (Dojindo Laboratories, Kumamoto, Japan)) for 35 min. After removal of the culture medium and washing with PBS, mitophagy-specific fluorescence was evaluated using a cell scoring module of MetaXpress^®^ Image Acquisition and Analysis Software. Data are expressed as percentage of control.

### 4.13. Lysosomal Proteolytic Activity Assay

Control and zinc pyrithione-treated cells of explant human melanoma grown in 96-well plates were at 1 h before analysis exposed to 10 µM DQ-Green BSA. They were then rinsed in warm cultivation medium, and the fluorescence originating from DQ-Green BSA cleavage in the functional lysosomes was measured fluorimetrically (TECAN SpectraFluorPlus, TECAN Austria GmbH, Grödig, Austria). Results were expressed as a time-dependent increase in fluorescence in arbitrary units.

### 4.14. Lysosomal Membrane Assay

Explant human melanoma cells (in the presence or absence of zinc pyrthione) were stained with 5 μM acridine orange for 15 min at individual treatment intervals. Cells were then rinsed twice with fresh cultivation medium, and acridine orange redistribution was measured fluorimetrically (TECAN SpectraFluorPlus, TECAN Austria GmbH, Grödig, Austria). Lysosomal membrane damage was expressed as an increase in diffuse cytosolic green fluorescence by acridine orange released from lysosomes in arbitrary units.

### 4.15. Gene Knockdown

Knockdown of Atg5 in explant melanoma cells was carried out using the siRNAs transfection kit from Santa Cruz Biotechnology, Inc. (Santa Cruz, CA, USA) following the manufacturer’s protocol. Human explant melanoma cells were seeded into six well tissue culture plates and cultivated until 75% confluence. The Atg5-specific siRNA duplex solution was mixed with transfection reagent, incubated for 45 min and then the cells were washed once with siRNA transfection reagent and exposed to a mixture of siRNA transfection medium with transfection reagent containing Atg5-specific siRNA duplex solution. The cells were incubated for 8 h at 37 °C. Next, 1 mL of standard cultivation medium was added to cells and incubation continued for another 24 h. In parallel, transfection of cells with scrambled Atg5-specific siRNAs sequence was carried out to provide negative control. Thus, transduced cells (untreated or exposed to zinc pyrithione) were then analyzed. Silencing of Atg5 was confirmed by immunoblotting analysis. Results were normalized to a mock control.

### 4.16. Cell Damage/Death

Control and zinc pyrithione-treated cells of explant human melanoma in plastic tissue-culture dishes with glass bottom were observed in a time-lapse imaging system BioStation IM (Nikon, Prague, Czech Republic). Recording was carried out in a both multipoint and multichannel time-lapse modes and upon a range of magnifications. Obtained sequences were software (NIS Elements AR 3.20 (Nikon, Prague, Czech Republic)) processed and analyzed with selection of typical frames depicting morphology of individual cells at the particular time intervals. The rate of cell damage/death was expressed as percentage of cells with changed morphology as compared to control cells appearance.

In addition, cleaved caspase-3 and PARP-1 positivity was detected in explant melanoma cells using the procedure detailed in the autophagy detection (see above) with the primary antibodies anti-cleaved caspase-3 (1:100) and anti-PARP-1 (1:100) and secondary FITC-conjugated goat anti-mouse.

### 4.17. Immunoblotting

Treated and control human explant melanoma cells were washed with PBS and harvested at different time intervals in ice-cold lysis buffer (150 mM NaCl, 10% glycerol, 1% n-octyl-β-D-glucopyranoside, 1% Triton X-100, 50 mM NaF, 50 mM Tris/HCl, 2 mM EDTA, 2 mM EGTA, 50 mM NaF, 1 mM sodium orthovanadate, Complete TMMini). The cell lysates were boiled for 5 min/95 °C in SDS sample buffer (Tris-HCl pH 6.81, 2-mercaptoethanol, 10% glycerol, SDS, 0.1% bromphenol blue) and loaded onto a 10% SDS/polyacrylamide gel. Each lysate contained equal amount of protein (15 μg) as determined by BCA assay. After electrophoresis, proteins were transferred to a PVDF (Polyvinylidene fluoride) membrane (200 V, 75 min) and blocked (1 h, at 25 °C) with 5% nonfat dry milk in TBST (Tris-Buffered Saline plus 0.05%Tween-20). Membranes were incubated with primary antibodies (monoclonal rabbit anti-Atg5, 1: 1000, monoclonal mouse anti-metallothionein IIA, 1:500, monoclonal mouse anti-β-actin, 1:100) at 4 °C overnight followed by five 6 min washes in TBST. Next, the blots were incubated with secondary peroxidase-conjugated antibodies (1:1000, 1 h, 25 °C), washed with TBST and the signal was developed with a chemiluminescence (ECL) detection kit (Boehringer Mannheim-Roche, Basel, Switzerland). β-actin was used as the loading control. Density of protein-specific signals was evaluated by GelQuant 2.7 software (DNR Bio-Imaging Systems, Jerusalem, Israel).

### 4.18. Statistics

Statistical analysis was carried out with the statistical program GraphPad Prism (GraphPad Software version 6.0, Inc. San Diego, CA, USA). We used a one-way ANOVA test with Dunnett’s post-test for multiple comparisons. Results were compared with control samples, with means considered significant at *p* < 0.05.

## Data Availability

Data is contained within the article.
